# Exploring the influence of deforestation on dengue fever incidence in the Brazilian Amazonas state

**DOI:** 10.1371/journal.pone.0242685

**Published:** 2021-01-07

**Authors:** Alexandra Kalbus, Vanderson de Souza Sampaio, Juliane Boenecke, Ralf Reintjes

**Affiliations:** 1 Department of Health Sciences, Faculty of Life Sciences, Hamburg University of Applied Sciences, Hamburg, Germany; 2 Fundação de Vigilância em Saúde do Amazonas, Manaus, Brazil; 3 Fundação de Medicina Tropical Dr. Heitor Vieira Dourado, Manaus, Brazil; 4 Programa de Pós-graduação em Medicina Tropical, Universidade do Estado do Amazonas, Manaus, Brazil; 5 Programa de Pós-graduação em Ciências da Saúde, Universidade Federal do Amazonas, Manaus, Brazil; University of Oklahoma Norman Campus: The University of Oklahoma, UNITED STATES

## Abstract

**Introduction:**

Dengue fever is the most prevalent arboviral disease in the Brazilian Amazon and places a major health, social and economic burden on the region. Its association with deforestation is largely unknown, yet the clearing of tropical rainforests has been linked to the emergence of several infectious diseases, including yellow fever and malaria. This study aimed to explore potential drivers of dengue emergence in the Brazilian Amazon with a focus on deforestation.

**Methods:**

An ecological study design using municipality-level secondary data from the Amazonas state between 2007 and 2017 (reported rural dengue cases, incremental deforestation, socioeconomic characteristics, healthcare and climate factors) was employed. Data were transformed according to the year with the most considerable deforestation. Associations were explored using bivariate analysis and a multivariate generalised linear model.

**Results:**

During the study period 2007–2017, both dengue incidence and deforestation increased. Bivariate analysis revealed increased incidences for some years after deforestation (e.g. mean difference between dengue incidence before and three years after deforestation was 55.47 cases per 100,000, p = 0.002), however, there was no association between the extent of deforestation and dengue incidence. Using a negative binomial regression model adjusted for socioeconomic, climate and healthcare factors, deforestation was not found to be related to dengue incidence. Access to healthcare was found to be the only significant predictor of dengue incidence.

**Discussion:**

Previous research has shown that deforestation facilitates the emergence of vector-borne diseases. However, no significant dose-response relationships between dengue incidence and deforestation in the Brazilian Amazonas state were found in this study. The finding that access to healthcare was the only significant predictor of dengue incidence suggests that incidence may be more dependent on surveillance than transmission. Further research and public attention are needed to better understand environmental effects on human health and to preserve the world’s largest rainforest.

## Introduction

Dengue fever is a mosquito-borne viral disease, caused by the four dengue virus serotypes of the family *Flaviviridae* (DENV1–4) [1]. Approximately 20% of infected humans show clinical manifestations which range from mild febrile illness to severe and fatal health complications [2, 3].

DENV is maintained in a transmission cycle between mosquitoes and, most commonly, humans [4]. The most prominent vector of DENV is the female *Aedes* mosquito. *Ae*. *aegypti*, regarded as the primary dengue vector, is highly adapted to urban living environments, whereas another well-known vector species, *Ae*. *albopictus*, is mainly abundant in peri-urban and rural areas [5, 6]. Both vectors are widespread in the tropics and subtropics, with *Ae*. *albopictus* also emerging in temperate zones [7].

Dengue is endemic in more than 100 countries throughout the tropics and subtropics [8], putting 3.83 billion people at risk and resulting in 390 million infections annually of which 96 million manifest symptomatically [9, 10]. In the Americas, the highest absolute numbers of dengue cases have been reported from Brazil throughout the last two decades [11]. In 2019, Brazil reported its highest number to date of more than 2.2 million suspected cases (1,038.4 cases per 100,000), compared to 2018 (124.9 per 100,000), 2017 (119.3 per 100,000) and 2016 (716 cases per 100,000) [11].

Climatic conditions known to influence the emergence of autochthonous dengue transmission include temperature, humidity, and rainfall [12–15]. Specifically, warm and wet climates create suitable living and breeding habitats for vector mosquitoes that further affect mosquito growth and length of the gonotrophic cycle, as well as the extrinsic DENV infection period (EIP) [16–19]. In suitable climates, main drivers of dengue disease are urbanisation, globalisation and inefficient mosquito control [20]. Urbanisation, especially when rapid and unplanned, may favour the establishment and further expansion of *Aedes* mosquitoes into urban settings in close proximity to human settlements [20]. Within these settings, low socioeconomic conditions have been associated with increased dengue transmission, in particular insufficient water supplies resulting in water storage, inadequate sewage, and garbage management [21–23].

Moreover, the access to healthcare services may shape the local dengue burden in two regards: on the one hand, more cases are detected in areas with sufficient healthcare capacities, resulting in rigorous dengue surveillance and thus higher incidence. On the other hand, high-quality care may reduce case fatalities resulting from severe dengue infections from over 20% to less than 1% [24]. As argued by Carabalí and Hendrickx, an underperforming healthcare system may lead to low and biased estimates of dengue incidence [25].

In addition to these determinants, there is increasing evidence that deforestation facilitates the transmission of certain infectious diseases through affecting the vector ecology [26, 27]. Despite the immense importance of tropical rainforest for the Earth’s climate and biodiversity, much of it is lost due to deforestation, i.e. conversion of forest to another land use [28, 29]. The main drivers of deforestation are agricultural expansion, urban growth, infrastructure development, and mining [30]. In the Brazilian Amazon, most anthropogenic land cover changes and degradations occur in the southern and eastern areas, referred to as the "arc of deforestation" [31]. Between 2007 and 2017, a total of 81,965 km^2^ forest was lost in the Legal Amazon (average per year = 7,451 km^2^) [32]. The greatest annual loss (12,911 km^2^) was reported in 2008, the lowest (4,571 km^2^) in 2012. From 2017 to 2018, 7,900 km^2^ of forest was cleared in the Legal Amazon, 13.7% more than the preceding year. In this period, the Brazilian state of Amazonas lost 1,045 km^2^ of forest land, corresponding to an increase in deforestation of 4.4% compared to the previous 12 months and 13.2% of the total deforestation in the Legal Amazon [32].

Interestingly, deforestation in the Amazon has been linked to vector-borne malaria, Lyme disease, and yellow fever transmission [33–37]. However, the effects of deforestation may vary for different diseases, including dengue, depending on disease ecology and transmission cycles. There have been few studies investigating the relationship between deforestation and dengue fever. Nakhapakorn and Tripathi found built-up and agricultural areas to be of high and moderate risk for dengue, respectively, compared to forested areas in Thailand [38]. Another study which investigated drivers of dengue fever in Indonesia 2006–2016 revealed a strong negative association between forest cover and dengue fever. The risk of dengue fever decreased by 9% (95% CI 8.5–9.5%) with a 1% increase in forest cover [39].

To date, no association between deforestation and dengue could be determined in the Amazon. Saccaro and colleagues assessed deforestation's impact on several infectious diseases and accidents caused by venomous animals in the Legal Amazon 2004–2012 [40]. They found that deforestation led to an increase in the incidences of visceral and subcutaneous leishmaniasis and malaria, but did not affect dengue fever. In addition, Bauch and colleagues investigated the influence of ecosystem changes on infectious diseases in the Legal Amazon 2003–2006, with findings revealing no association between deforestation and dengue fever [41]. However, research exploring the relationship between deforestation and dengue fever is scarce. This research seeks to address this knowledge gap in exploring the association between deforestation and dengue incidence in the Brazilian state of Amazonas.

## Materials and methods

Amazonas is the largest state in Brazil, with a total area of 1,559,168.117 km^2^, and a total population of 4,080,611 in 62 municipalities in 2018 [42, 43]. The state is characterised by an equatorial climate, with both high temperatures and rainfall [44]. Between 2010 and 2015, mean annual temperature ranged from 23.43°C to 26.24°C with no clear trend indicated. Accumulated rainfall was on average 2149 mm per year, with a maximum of 3023 mm in 2011 and a drop to 1178 mm in 2015. Relative humidity averaged at 93.59% and displayed an increasing trend until 2014, when a maximum of 97.04% was followed by a drop to 89.61% in 2015.

### Data

Because dengue is known as an urban disease [3, 45], whereas deforestation occurs outside from cities, this research focused on rural communities. The units of analysis were municipalities and years. Data on dengue cases per municipality from 2007 to 2017 were retrieved from SINAN, Brazil’s Notifiable Disease Information System (http://portalsinan.saude.gov.br/). Cases included all records of reported cases of classical dengue fever, dengue fever with complications, dengue haemorrhagic fever or dengue shock syndrome. All classifications followed the corresponding recommendations of the World Health Organization (WHO) [46]. Cases were furthermore differentiated in rural, peri-urban and urban, according to the classification by the Brazilian Institute of Geography and Statistics (IBGE) [47]. Peri-urban cases were combined with rural cases, amounting to 5,077 (2.96%) of the 164,671 valid cases. Three municipalities were found to have incomplete records: Pauini had no record for 2007–2013, Tonantins and Nhamundá had no records for 2014–2018. For these, the available years were treated as the study period. Inclusion criteria of cases were the presence of information about urban/rural status and their respective municipality. Sizes of the rural population per municipality were derived from the 2000 and 2010 IBGE population censuses [48] and determined for the years in between and after 2010 using linear inter- and extrapolation. Dengue fever incidence was then calculated per 100,000 population. Population estimates for each municipality were assessed for completeness and consistency, with only the municipality Iranduba indicating inconsistent estimates. Due to a sharp decrease in population size from 2000 to 2010, extrapolation yielded very small to negative population counts resulting first in overestimating and then negative incidences. Hence, the municipality was excluded from the analysis.

For the purpose of this study, deforestation is described as the incremental forest loss from one year to another (in km^2^) and as a proportion of the total forest area of the preceding year. Deforestation per municipality was obtained from the Project for Monitoring Deforestation in the Legal Amazon (PRODES) by the National Institute for Space Research (INPE) [49]. Relative deforestation rates compared to the 2007 forest area were calculated.

The following socioeconomic indicators were included: Mean monthly household income per capita, the proportion of poor population and population living in households with semi-adequate sanitation [48], and the Municipal Human Development Index (MHDI) [50]. Except for the last, all indicators refer to the rural population. To approximate the likelihood of case notification through the healthcare system, the Performance Index of the Unified Health System (Índice de Desempenho do SUS [IDSUS]) was included in the study [51]. The IDSUS includes 24 indicators to assess the potential access, the access obtained, and the effectiveness of health system services. Access to and effectiveness of healthcare were included in the analysis. The variable access to healthcare encompasses 16 indicators of basic health assistance, reflecting both the potential and actually obtained access to the public health system. The effectiveness variable describes the performance of the public health system through eight indicators. Both variable indicators range from 0 to 10, with higher values indicating a better performance [52]. As climatic conditions are known to influence DENV transmission [10], mean annual temperature (°C), relative humidity (%) [53], and annual cumulative precipitation (mm) [54] for 2010–2015 were included. S1 Table provides a detailed variable description.

For each municipality, the year with the highest absolute forest loss was determined to define deforestation events for further analysis. That way, dengue incidences one year before and 1–5 years after deforestation were compared. If the year with the highest absolute forest loss did not meet one or more of the following inclusion criteria, the year with the next most significant loss was selected: (a) Dengue incidence data were available in the years following the deforestation event, (b) the latest possible year of a deforestation event was determined by the respective outcome (incidence 1–5 years later, i.e. 2016 was the latest possible year for incidence after one year), (c) for analyses that entail the comparison of dengue incidence before and after deforestation, the earliest possible year of deforestation was 2008, and (d) if two or more years which all meet the above criteria present the same, greatest absolute forest loss, then the later year was considered.

The authors employed exclusively historical secondary, publicly available data for the present analysis. Thus, no ethical clearance was required.

### Statistical analysis

Statistical analysis was performed using R (packages dplyer, ggplot2, sandwich, AER, MASS) [55–59]. Significance level for all statistical tests was set to p < 0.05.

Baseline information was presented on all studied variables. The influence of deforestation on dengue incidence was first approached descriptively.

Bivariate analyses comprised Pearson correlation and paired samples *t*-tests. Pearson correlation analyses were used to determine associations of all study variables with mean rural dengue incidence. Such tests were also performed with the transformed data to test for possible dose-response relationships between deforestation and dengue incidence 1–5 years later, respectively. These tests were furthermore stratified according to whether a dengue outbreak took place (2011 & 2014) the year before and the respective year after the year of the greatest deforestation event. Dependent *t*-tests were conducted to determine whether there was a difference between dengue incidence before and after the deforestation event. Five tests using the transformed data were performed comparing the mean dengue incidence before the deforestation event with the incidence of 1–5 years after the deforestation event. A subsequent analysis comprised five paired samples *t*-tests of dengue incidences with a lag of 2–6 years around randomly selected years. These tests were performed to explore a potential underlying time trend which may affect observed effects when assessing the changes in incidence in the years following a deforestation event.

Finally, multivariate generalised linear models were performed to assess how the distribution of mean dengue incidence is affected by the extent of deforestation during the study period while controlling for other variables. As dengue incidence was presented as count data and followed a Poisson-like distribution, a Poisson regression model approach was considered suitable for the analysis. However, due to significant overdispersion (variance unequal to the mean), modification of the Poisson regression model was required. Two common ways to address over-dispersed count outcome variables are quasi-Poisson and negative binomial regression. Because the relationship between mean and variance corresponds to a negative binomial (non-linear relationship) rather than quasi-Poisson distribution (linear relationship), a negative binomial regression approach was chosen. The selection of an appropriate prediction model was based on an exploratory analysis using different subsets of variables, including the assessment of the model fit with and without the predictor variables. Model selection criteria included AIC and BIC, the total number of predictor variables as well as the number of significant predictor variables included in the model, considering the theoretical context of this research. The chosen model had the lowest possible AIC as well as BIC value with an adequate number of relevant environmental and sociodemographic predictor variables to be included in the analysis (mean relative forest loss, MHDI, access to healthcare and mean annual temperature).

## Results

A description of the study variables is given in Table 1. The mean MHDI was 0.57, reflecting a low development. The mean monthly household income per capita was R$147.6 (≈ US$84), while 87.8% of the population was considered poor. 14.7% had semi-adequate sanitation facilities. Amazonas had an average temperature of 24.9°C, mean annual precipitation of 2149 mm, and 93.6% mean relative humidity. The effectiveness of healthcare index was 7.95 on average, corresponding to a good performance, whereas the access to healthcare index was less (mean = 2.95).

**Table 1:** Description of the study variables.

**Variable**	**n**	**Mean (± SD)**	**Median**	**Min**	**Max**
Sociodemographic Characteristics					
**Municipality Human Development Index**	62	0.57 (± 0.05)	0.56	0.45	0.74
**Household income per capita (R$) per month**	62	147.6 (± 56.37)	132.0	73.0	357.0
**Proportion (%) of poor population**	62	87.81 (± 7.15)	89.21	60.01	96.21
**Proportion (%) of population with semi-adequate sanitation**	62	14.72 (± 12.05)	11.26	0.36	53.18
**Dengue Incidence**					
**Mean incidence of dengue fever per 100,000**	58	57.84 (± 107.02)	16.54	0.00	636.90
**Deforestation**					
**Mean annual forest loss (km**^**2**^**)**	62	10.09 (± 23.13)	3.09	0.41	139.34
**Mean annual forest loss (%)**	62	0.089 (± 0.165)	0.032	0.002	1.121
**Total loss of forest area (km**^**2**^**)**	62	110.97 (± 254.42)	33.90	4.50	1532.70
**Total forest loss (% of 2007 area)**	62	0.98 (± 1.81)	0.36	0.02	12.33
**Climatic and Environmental Factors**					
**Temperature (°C)**	62	24.88 (± 0.63)	25.08	23.43	26.24
**Precipitation (mm)**	61	2149 (± 365.24)	2113	1178	3023
**Relative humidity (%)**	62	93.59 (± 1.27)	93.64	89.61	97.04
**Healthcare Indicators**					
**Access to healthcare**	62	2.95 (± 0.80)	3.00	1.38	4.71
**Effectiveness of healthcare**	62	7.95 (± 0.53)	7.93	6.86	9.07

SD = Standard deviation

**Fig 1:**
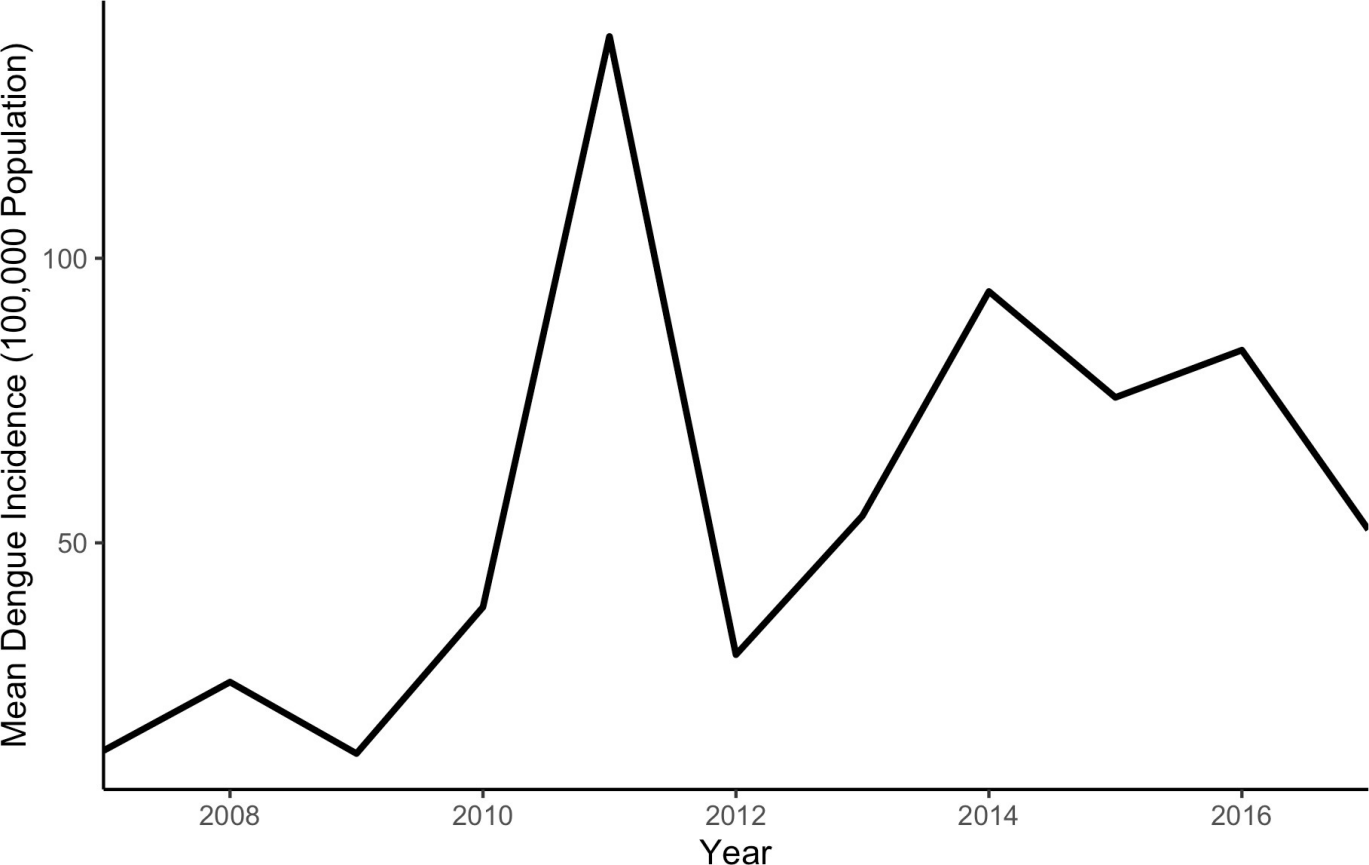
Dengue incidence per 100,000 in Amazonas 2007–2017.

The average annual incidence of reported rural dengue cases in Amazonas was 57.84 per 100,000 during the study period. A mean of zero rural cases was reported from the municipalities Amaturá, Eirunepé, Envira, and Santa Isabel do Rio Negro, whereas Manaus reported the highest average incidence (636.9 per 100,000). Average dengue incidence was 13.49 cases per 100,000 rural population in 2007, increased in 2010 and peaked in 2011 at 138.97 cases per 100,000 (see Fig 1). After a decrease in 2012, incidence again reached a peak in 2014 which was lower than in 2011 (94.74 cases per 100,000). Since then, incidence displayed a decreasing trend with an especially steep drop to 52.31 cases per 100,000 rural population in 2017.

**Fig 2:**
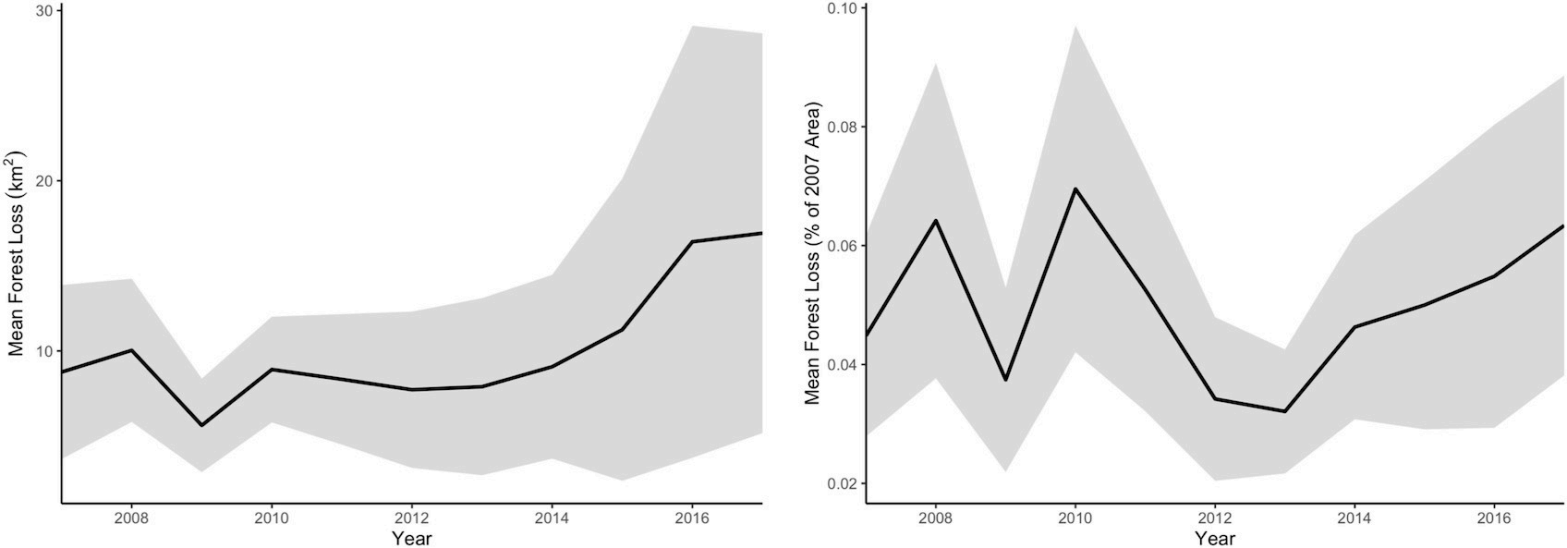
Annual forest loss in Amazonas 2007–2017 (left: km^2^, right: % of 2007 area; shaded areas indicate 95% confidence intervals).

In 2007, an average of 77.79% of the municipalities' surface was covered by forest. Ipixuna had the highest forest cover with 97.30%, Urucurituba the lowest with 4.51%. The most significant absolute total loss during the study period occurred in Lábrea (1532.7 km^2^), the greatest relative total loss in Careiro da Várzea (12.3%). The average absolute and relative deforestation during the study period was 10.1 km^2^ and 0.09%, respectively. Fig 2 displays the trajectories of absolute and relative forest loss during the study period. The average absolute annual forest loss in Amazonas was 8.8 km^2^ forest loss per municipality in 2007, with its low of 5.6 km^2^ in 2009. Since 2012 (7.7 km^2^), incremental absolute deforestation on the municipality level increased up to 16.9 km^2^ in 2017, about twice the deforestation levels from 2007. Incremental relative deforestation, i.e. the loss compared to the area of the preceding year, was 0.04% on average per municipality. The highest relative deforestation on the municipality level followed an initial peak in 2008 (0.06%) during the study period, with 0.07% forest loss per municipality in 2010. Since then, the deforestation rate declined to 0.03% in 2013, with an upward trend to 0.06% until 2017.

When assessing the mean annual dengue incidence and deforestation rates in the study region, no similar patterns of rural dengue incidence could be described. However, municipalities in the central region tend to have higher incidences, while municipalities in the south and east of Amazonas experienced more significant deforestation than the rest of the state. S2 Table provides the mean dengue incidence per 100,000 and mean deforestation rate during the study period 2007–2017 for each municipality.

The greatest forest loss per municipality was 75% higher on average than the mean annual deforestation during the study period. S3 Table shows the highest forest loss compared to the mean annual loss throughout the study region.

Most municipalities experienced an increase in incidence during the years following deforestation. S4 Table shows the differences between the dengue incidence one year before, and the incidence one, two, three, four, and five years after deforestation per municipality. Overall, later years after deforestation tend to present stronger increases in dengue incidence.

Pearson correlation tests between average annual dengue fever incidence and the chosen study variables revealed significant associations of dengue incidence with MHDI (r = 0.51, p<0.001), the proportion of poor population (r = -0.28, p = 0.034), the average monthly household income (r = 0.33, p = 0.012), and the access-related performance of healthcare systems (r = 0.45, p<0.001) (Table 2). No climatic variable, and neither absolute nor relative average annual forest loss, correlated significantly with average dengue incidence. The extent of neither forest loss variable appeared to be associated with dengue incidence one to five years after the deforestation event. Stratification for outbreaks occurring in the respective year after deforestation (2011, 2014) yielded similar results. Furthermore, access to healthcare was positively associated with the household income per month (r = 0.441, 95% CI 0.215–0.622, p<0.001), MHDI (r = 0.638, 95% CI 0.462–0.766, p<0.001) and inversely associated with the proportion of poor population (r = -0.394, 95% CI -0.586–-0.160, p = 0.002).

**Table 2:** Pearson correlations of mean dengue incidence with study variables.

Variable	n	Pearson r	p
**Municipality Human Development Index**	58	0.512	<0.001*
**Household income per capita (R$) per month**	58	0.328	0.012*
**Proportion (%) of poor population**	58	-0.279	0.034*
**Proportion (%) of population with semi-adequate sanitation**	58	0.234	0.076
**Annual forest loss (km**^**2**^**) during the study period**	58	0.029	0.829
**Annual forest loss (%) during the study period**	58	-0.047	0.724
**Temperature (°C)**	58	-0.009	0.948
**Precipitation (mm)**	57	-0.184	0.170
**Relative humidity (%)**	58	-0.031	0.816
**Access to healthcare**	58	0.449	<0.001*
**Effectiveness of healthcare**	58	0.044	0.744

* significance on the p < 0.05 level

Paired samples *t*-tests demonstrated significant mean differences of the dengue incidence one year before deforestation and two years (31.27 per 100,000, p = 0.004), three years (55.47 per 100,000, p = 0.002), and five years after deforestation (67.71 per 100,000, p = 0.035) (Table 3). All tests with randomly selected years except for one showed no significant differences, indicating the absence of an underlying secular trend.

**Table 3:** Paired samples *t*-tests for dengue incidence 1 year before and 1–5 years after deforestation and corresponding random years.

	N	Mean of the differences	95% CI	t	df	p
**1 year after deforestation**	57	56.64	-34.26–147.55	1.25	56	0.217
**Random 2-year time lag**	61	65.52	-4.39–135.43	1.87	60	0.066
**2 years after deforestation**	59	31.27	10.21–52.22	2.99	58	0.004*
**Random 3-year time lag**	61	16.77	-41.32–74.86	0.58	60	0.566
**3 years after deforestation**	59	55.47	20.60–90.35	3.18	59	0.002*
**Random 4-year time lag**	58	41.63	1.28–81.97	2.07	57	0.043*
**4 years after deforestation**	58	57.60	-0.90–116.10	1.97	57	0.054
**Random 5-year time lag**	60	18.13	-18.09–54.35	1.00	59	0.321
**5 years after deforestation**	58	67.71	4.78–130.63	2.15	57	0.035*
**Random 6-year time lag**	60	27.26	-6.63–61.15	1.61	59	0.113

* significance on the p < 0.05 level

Given the results of the multivariate negative binomial regression analysis, access to healthcare was found to be the only covariate coefficient significantly different from zero. The parameter estimates and standard errors for the variables included in the final model are given in Table 4. Mean annual forest loss, MHDI and mean annual temperature were no significant predictors of dengue incidence. Access to healthcare was found to be significantly and positively related to dengue (Estimate = 0.691, SE = 0.246, p = 0.005).

**Table 4:** Parameter estimates for covariates.

Covariate	Coefficient estimate	Standard error	95% Confidence Interval	Prob>|z|
**Mean annual forest loss (%)**	-1.136	0.919	-2.938–0.666	0.217
**MHDI**	0.511	0.306	-0.088–1.110	0.095
**Access to healthcare**	0.691	0.246	0.208–1.174	0.005*
**Mean temperature**	-0.055	0.283	-0.608–0.498	0.845

*significance on the p < 0.05 level.

## Discussion

This study aimed to explore the rural dengue fever incidence in relation to deforestation in Amazonas between 2007 and 2017. During the study period, dengue incidence followed an overall increasing trend, with peaks in 2011 and 2014 and a slight decrease in 2017. The municipalities with the highest dengue fever burden (Manaus, Guajará and Tefé) appear to be scattered throughout the state. Absolute forest loss increased considerably since 2013, while relative forest loss reached its highest level in 2010 and, after a sharp decrease, demonstrated a steep increase since 2013. Most affected municipalities are located in the south and east of Amazonas. The comparison of dengue incidences before and one to five years after major deforestation events indicated increases in incidence two, three, and five years after deforestation. The comparison of incidences with the same lag times independently from deforestation by selecting years randomly did not reveal significant differences, indicating an absence of underlying time trends. However, when assessing the influence of higher deforestation levels on increasing dengue fever incidence during the study period while controlling for other variables, no association could be found. Instead, the findings of the analysis reveal that access to healthcare was the only significant predictor of dengue incidence. Access to healthcare was also found to be associated with socioeconomic markers, namely MHDI, proportion of poor population and income, all of which were correlated with dengue incidence in the bivariate analysis.

In order to verify the trends seen in the historical health data derived for Amazonas, the study data were compared to the epidemic trends seen in the American region. Brazil and other Latin American countries faced dengue fever epidemics in 2010/2011 and 2015/2016 [60]. The 2010/2011 epidemic is also recorded in the Amazonas data, mainly in 2011, whereas a second outbreak in 2014 can rather be suspected. A drop in the 2017 dengue incidence is in line with reports on the American region provided by the WHO [24], with different potential reasons debated in the literature. In 2015/2016, Zika virus (ZIKV, *Flaviviridae* family) caused a major epidemic in the Americas, which the WHO declared a Public Health Emergency of International Concern in early 2016. Like DENV, ZIKV shares the same mosquito vector *Ae*. *aegypti* [61], shows a similar clinical picture and complicates accurate DENV serological diagnostics because of serological cross-reaction with other flaviviruses [62–64]. Moreover, Rico-Mendoza *et al*. concluded that a decrease in dengue cases seen in Colombia after co-circulation of dengue, Zika and chikungunya viruses could yield cross-protection against each of the *Aedes*-borne viruses [65]. On the backdrop of the Zika outbreak in Brazil in 2015/2016 [66], the impact of cross-protection but also cross-reactivity in diagnostic testing should be investigated further to understand the epidemiological trends of co-circulating DENV and ZIKV.

A similar comparative approach was followed for the deforestation data. This study points to the south and south-east of Amazonas to experience most deforestation. These locations correspond to Amazonas’ borders to Pará, Mato Grosso and Rondônia, all known as states with high deforestation levels [32].

Within the context of this study, the findings support the results of Nakhapakorn et al., who found no association between deforestation and dengue fever in Thailand [38], as well as Saccaro et al., who used PRODES data to examine the effect on deforestation on multiple infectious diseases across the Legal Amazon [40]. However, the association of healthcare access and dengue incidence as found in this study may point to an important limitation when using historical health report data available for Amazonas. Reported dengue incidence may be considerably more dependent on the capacities and quality of the reporting system than the real disease burden, as dengue data presented through passive surveillance are known to be subject to underreporting and inequality [25, 67, 68]. The associations of the socioeconomic indicators MHDI, proportion of poor population and income with access to healthcare identified in this study further suggest that patterns in reported dengue incidence are primarily driven by surveillance rather than transmission dynamics. There is a need for more comprehensive analysis and field research to better understand the relationship between deforestation events and dengue transmission.

### Limitations

This study has potential limitations concerning the data and methods employed.

Choosing an ecological study design with aggregated data poses a potential threat of ecological fallacy and impedes the consideration of population dynamics such as age as well as interannual climatic variations, both relevant for DENV transmission [67, 68]. There may further be disparities in the timely fit of data when using deforestation data (assessed mid-year), census data (2010 reports), and climatic data (only available for 2010–2015).

Furthermore, there is potential inaccuracy in deforestation levels reported through the forest monitoring system. Richards et al. compared the deforestation of Amazon rainforest for the years 2008–2012 captured by PRODES with the forest loss captured by remotely-sensed datasets (Global Forest Change dataset and Fire Information for Resource Management System) [69] and found considerable divergence with an estimated 9,000 km^2^ forest loss that was not captured by PRODES, especially in Pará, Mato Grosso and Rondônia. In Amazonas, the highest divergence was found in the north-east, which corresponds to the areas of highest relative forest loss found in this study (see S2 Table). With the overall divergence being low in this state, deforestation can be interpreted with reasonable confidence, bearing in mind that actual deforestation in the most-affected areas was higher than the data suggest.

Finally, this study investigated the influence of deforestation on dengue cases in rural settings, which is less than 3% of all dengue cases in Amazonas. Hence, generalising conclusions from these findings must be drawn with caution as rural dengue incidence only a small proportion of the dengue burden in Amazonas.

This research presents the following methodological limitations: First, the difference in dengue incidence following deforestation events resulting from *t*-tests could be a consequence of an underlying trend rather than a direct effect of deforestation. Although testing randomly selected years with corresponding time lags did not suggest any underlying trends, other environmental or socioeconomic factors may influence the observed effects. Another limitation of the *t*-tests is the selected baseline year, which was compared with the year after the primary deforestation event and was set as the year prior to the event. Due to interannual variability in dengue incidence levels, the baseline levels could be considerably higher or lower than in other years, hence biasing the comparison with later years after the deforestation event. Second, overdispersion seen in the Poisson model, which was addressed by a negative binomial approach, could indicate the absence of important other influencing factors. Those include, for example, the implementation of prevention and mitigation strategies, the presence of different DENV serotypes throughout the study area, population immunity levels, urbanisation across municipalities or fine-scale climate information, which were not accessible for this study. Incorporating further information in an advanced statistical framework could enhance our understanding of dengue fever distribution in Amazonas and its link to deforestation. Third, the variables chosen in this study are mean incidence and mean deforestation rates, which do not account for trends over time. An approach with finer time scales would allow for a more comprehensive understanding of potential associations between deforestation events and temporal changes in dengue incidences.

Despite these limitations, this study entails a novel approach to avoid the bias of investigating urban disease epidemiology associated with a rural explanation. Urban populations are mostly in no contact with forests (and hence, deforestation), yet suffer from a higher dengue burden than rural populations [24]. Focusing the analysis on rural and peri-urban dengue transmission areas corresponds to deforestation, as these populations are in closer proximity to forests, and thus more likely to be affected by changes to the sylvatic environment than urban populations. To the authors’ best knowledge, this is the first analysis of the effect of deforestation on dengue fever in Amazonas, Brazil, using an ecological study design that excludes urban incidence numbers. Moreover, the comprehensive approach of this study is of significant advantage, as most research performed on the burden of dengue fever and other mosquito-borne diseases focused solely on climatic factors or climatic and sociodemographic influences [15, 17, 70]. Multiple perspectives need to be considered to explore disease dynamics more profoundly, including climate, socioeconomics, urbanisation, and vector distribution [71].

### Implications of the study and directions for future research

This study could not identify a link between deforestation and dengue fever incidence in the rural areas of the Brazilian Amazon. However, deforestation could be linked to other vector-borne infectious diseases in the Amazon, such as malaria, leishmaniasis and yellow fever [43, 72]. They are complemented by other emerging diseases of public health relevance which have been linked to deforestation, including Oropouche fever, the second most prevalent arboviral disease in Brazil, maintained in both an urban and sylvatic transmission cycles [73, 74], and Mayaro fever, which is usually vectored by forest mosquitoes but can also be transmitted by *Aedes* mosquitoes [75].

Due to the presence of sylvatic DENV transmission cycles in Africa and Asia, deforestation may have a strong impact on disease transmission in these regions [76]. Given that there is little to no adaptive barrier of sylvatic strains to infect humans, new DENV strains could add to the current burden, maintaining the disease even if tetravalent vaccines would be implemented [77]. Integrated vector control and effective surveillance are thus crucial to control these diseases on the backdrop of increasing deforestation rates.

The findings of this study serve as a starting point for further research by highlighting potentials and challenges as well as indicating areas of future research. Key challenges of an ecological study design are a lack of granularity and conflicts in data harmonisation, especially when using Open Data. More fine-scaled but sufficiently structured temporal and spatial information is needed to disentangle trends and associations of potential drivers of dengue emergence in the Brazilian Amazon, with a focus on deforestation. One possibility is to use fine-scale data, for example, on household levels and evaluate dengue incidence and deforestation levels within 200 m on a weekly basis. Data may then be analysed using a time-sensitive approach, such as time series analysis, that may be better suited to follow changes in dengue incidence and its relation to changes in deforestation rates over time. Another challenge in researching environmental influences is of a conceptual nature. Environmental factors are known to affect the distribution and behaviour of insect vectors, and to some extent, pathogen performance, which both shape disease dynamics in human populations [37]. As consistent mosquito surveillance data were not available, this analysis took advantage of proxy data of dengue transmission hotspots using reported dengue incidences. Deforestation can further be considered as an approximation because the land use subsequent to forest clearing (e.g. agriculture, settlements, infrastructure) is an important dengue risk factor [76]. For example, two studies evaluated the health effects of land uses in the Brazilian Amazon, including protected areas, indigenous reserves, roads, agriculture and mining [41, 78]. Although neither studied dengue fever, both constitute good examples for integrating land use in addition to deforestation levels in the research frame. Urbanisation, which may follow the clearing of forest, is thought to be a risk factor for dengue transmission [79]. This is mainly due to higher population density, increased contact among susceptible populations, and increasing sources of artificial water [20].

Thus, the following recommendations result from this study: Research should (1.) be conducted using more fine-scaled temporal and spatial data, (2.) incorporate data on the implementation of preventive and control measures, circulation of DENV serotypes and population immunity levels, (3.) consider multiple steps on the causal chain with special regard to land use following deforestation, and (4.) consider three indicators of DENV transmission: (a.) dengue incidence, (b.) mosquito abundance (e.g. surveillance in areas with and without deforestation), and (c.) DENV data (e.g. estimates of EIP before and after deforestation, or viraemia in infected mosquitoes, humans, and potential reservoir hosts).

## Conclusion

The consequences of deforestation are manifold and complex, and their effects reach from climate change and biodiversity to significant human health impacts. This study did not find an association of deforestation of tropical rainforest on dengue fever incidence in the Brazilian state of Amazonas. Although a potential link was indicated through the descriptive and bivariate analysis, a subsequent multivariate approach did not support these findings. The challenges of investigating their effect on dengue fever in the Brazilian Amazon were highlighted and recommendations for future research were derived. The more is known about the links between forest ecosystems and human health, and the better such knowledge is communicated, the better these forests can be protected.

## Supporting information

S1 Table Variable description. (DOCX)

S2 Table Mean dengue incidence and mean relative forest loss per municipality in Amazonas 2007–2017. (DOCX)

S3 Table Greatest forest loss compared to mean loss per municipality in Amazonas. (DOCX)

S4 Table Change in dengue incidence 1–5 years after deforestation event. (DOCX)
