# Supplementary material for: Exploring the influence of deforestation on dengue fever incidence in the Brazilian Amazonas state
Source: PLoS One. 2021 Jan 7;16(1):e0242685. doi: 10.1371/journal.pone.0242685 (PMC7790412; doi:10.1371/journal.pone.0242685)
Supplement: S1 Table — (DOCX) [file pone.0242685.s001.docx]

| **S1 Table. Variable description** | | |
| --- | --- | --- |
| Variable | Description | Source |
| Income | Mean household income per capita of the rural population, expressed in Reais (R$) in 2010 | IBGE population census, 2010 |
| Poor population | Proportion of individuals in the rural population with a per capita household income up to half of the minimum wage in 2010 (R$255.00 per month in August 2010) | IBGE population census, 2010 |
| Sanitation | Proportion of rural population living in households with semi-adequate sanitation (i.e. at least one of the following: water supply by general network, sewage by general network or septic tank, direct or indirect garbage collection) in 2010 | IBGE population census, 2010 |
| MHDI | Municipal Human Development, expressed as a value between 0 and 1, with 1 indicating a very high human development | United Nations Development Programme Brazil, Ipea and João Pinheiro Foundation, 2012 |
| IDSUS access | Subcomponent of the Performance Index of the Unified Health System (IDSUS) which assesses the potential and obtained access of healthcare systems on a scale between 0 and 10 | Brazilian Ministry of Health, N/A |
| IDSUS effectiveness | Subcomponent of the IDSUS which assesses the effectiveness of healthcare systems | Brazilian Ministry of Health, N/A |
| Temperature | Mean annual temperature between 2010 and 2015, measured 2 m above soil and expressed in °C | National Centers for Environmental Prediction (NCEP) |
| Relative Humidity | Mean annual relative air humidity in % | NCEP |
| Precipitation | Cumulative annual precipitation in mm between 2010 and 2015 | Unified Precipitation Project of the NOAA Climate Prediction Center |
