# Supplementary material for: Exploring the influence of deforestation on dengue fever incidence in the Brazilian Amazonas state
Source: PLoS One. 2021 Jan 7;16(1):e0242685. doi: 10.1371/journal.pone.0242685 (PMC7790412; doi:10.1371/journal.pone.0242685)
Supplement: S2 Table — (DOCX) [file pone.0242685.s002.docx]

| **S2 Table. Mean Dengue Incidence and Mean Relative Forest Loss per Municipality in Amazonas 2007–2017** | | |
| --- | --- | --- |
| Municipality | Mean dengue incidence per 100,000 (SD) | Mean relative forest loss (% of 2007 area) (SD) |
| Alvarães | 55.42 (67.19) | 0.06 (0.005) |
| Amaturá | 0.00 (0.00) | 0.03 (0.03) |
| Anamã | 13.30 (29.00) | 0.03 (0.03) |
| Anori | 28.06 (45.76) | 0.01 (0.01) |
| Apuí | 21.95 (23.07) | 0.17 (0.08) |
| Atalaia do Norte | 6.21 (7.62) | 0.00 (0.00) |
| Autazes | 9.38 (12.71) | 0.22 (0.13) |
| Barcelos | 26.73 (35.92) | 0.00 (0.00) |
| Barreirinha | 2.98 (3.43) | 0.09 (0.06) |
| Benjamin Constant | 10.48 (12.38) | 0.01 (0.01) |
| Beruri | 8.65 (14.93) | 0.01 (0.01) |
| Boa Vista do Ramos | 14.00 (28.71) | 0.03 (0.03) |
| Boca do Acre | 101.41 (124.16) | 0.23 (0.10) |
| Borba | 66.28 (90.91) | 0.01 (0.01) |
| Caaparinga | 7.95 (9.14) | 0.02 (0.02) |
| Canutama | 5.80 (10.88) | 0.09 (0.04) |
| Carauari | 6.53 (16.99) | 0.00 (0.00) |
| Careiro | 62.35 (68.62) | 0.06 (0.05) |
| Careiro da Várzea | 11.48 (8.30) | 0.33 (0.19) |
| Coari | 146.10 (151.86) | 0.01 (0.01) |
| Codajás | 34.33 (47.91) | 0.01 (0.01) |
| Eirunepé | 0.00 (0.00) | 0.02 (0.01) |
| Envira | 0.00 (0.00) | 0.11 (0.05) |
| Fonte Boa | 3.66 (9.25) | 0.01 (0.01) |
| Guajará | 348.65 (592.83) | 0.06 (0.03) |
| Humaitá | 131.25 (140.07) | 0.04 (0.03) |
| Ipixuna | 8.14 (11.94) | 0.03 (0.02) |
| Iranduba | *NA* | 0.15 (0.09) |
| Itacoatiara | 85.61 (76.26) | 0.13 (0.08) |
| Itamarati | 2.67 (8.87) | 0.01 (0.01) |
| Itapiranga | 11.37 (25.30) | 0.02 (0.01) |
| Japurá | 25.13 (55.13) | 0.00 (0.00) |
| Juruá | 3.56 (7.93) | 0.01 (0.01) |
| Jutaí | 11.49 (23.17) | 0.00 (0.01) |
| Lábrea | 56.72 (56.45) | 0.20 (0.14) |
| Manacapuru | 118.45 (116.58) | 0.07 (0.06) |
| Manaquiri | 11.87 (19.41) | 0.03 (0.02) |
| Manaus | 636.90 (703.26) | 0.03 (0.02) |
| Manicoré | 95.46 (124.81) | 0.12 (0.05) |
| Maraã | 24.14 (32.02) | 0.01 (0.01) |
| Maués | 23.25 (22.69) | 0.04 (0.02) |
| Nhamundá | *NA* | 0.03 (0.03) |
| Nova Olinda do Norte | 33.95 (39.97) | 0.05 (0.04) |
| Novo Airão | 25.50 (26.27) | 0.00 (0.00) |
| Novo Aripuanã | 79.89 (66.56) | 0.13 (0.11) |
| Parintins | 15.89 (22.84) | 0.07 (0.03) |
| Pauini | *NA* | 0.02 (0.01) |
| Presidente Figueiredo | 181.65 (154.58) | 0.04 (0.02) |
| Rio Preto da Eva | 53.03 (104.80) | 0.05 (0.03) |
| Santa Isabel do Rio Negro | 0.00 (0.00) | 0.00 (0.00) |
| Santo Antônio do Içá | 16.81 (44.86) | 0.01 (0.01) |
| São Gabriel da Cachoeira | 97.28 (141.57) | 0.00 (0.00) |
| São Paulo de Olivença | 0.55 (1.83) | 0.00 (0.01) |
| São Sebastião do Uatumã | 3.36 (7.56) | 0.03 (0.01) |
| Silves | 4.13 (19.9) | 0.04 (0.02) |
| Tabatinga | 164.85 (237.33) | 0.04 (0.03) |
| Tapauá | 16.28 (21.06) | 0.00 (0.00) |
| Tefé | 368.56 (386.08) | 0.02 (0.02) |
| Tonantins | *NA* | 0.02 (0.03) |
| Uarini | 2.03 (6.72) | 0.03 (0.03) |
| Urucará | 8.33 (27.67) | 0.01 (0.01) |
| Urucurituba | 44.90 (140.56) | 0.01 (0.01) |
