# Supplementary material for: Exploring the influence of deforestation on dengue fever incidence in the Brazilian Amazonas state
Source: PLoS One. 2021 Jan 7;16(1):e0242685. doi: 10.1371/journal.pone.0242685 (PMC7790412; doi:10.1371/journal.pone.0242685)
Supplement: S3 Table — (DOCX) [file pone.0242685.s003.docx]

| **S3 Table. Greatest Forest Loss Compared to Mean Loss per Municipality in Amazonas** | |
| --- | --- |
| Municipality | Greatest forest loss compared to mean loss (%) |
| Alvarães | 218.29 |
| Amaturá | 220.35 |
| Anamã | 66.70 |
| Anori | 171.92 |
| Apuí | 169.43 |
| Atalaia do Norte | 0.00 |
| Autazes | 109.07 |
| Barcelos | 0.00 |
| Barreirinha | 159.37 |
| Benjamin Constant | 276.92 |
| Beruri | 217.92 |
| Boa Vista do Ramos | 121.64 |
| Boca do Acre | 147.43 |
| Borba | 264.97 |
| Caaparinga | 266.51 |
| Canutama | 204.80 |
| Carauari | 231.73 |
| Careiro | 181.67 |
| Careiro da Várzea | 66.93 |
| Coari | 175.10 |
| Codajás | 115.35 |
| Eirunepé | 237.91 |
| Envira | 193.58 |
| Fonte Boa | 86.69 |
| Guajará | 159.45 |
| Humaitá | 123.18 |
| Ipixuna | 156.16 |
| Iranduba | 0.00 |
| Itacoatiara | 106.15 |
| Itamarati | 144.10 |
| Itapiranga | 133.81 |
| Japurá | 516.70 |
| Juruá | 237.74 |
| Jutaí | 190.40 |
| Lábrea | 198.52 |
| Manacapuru | 159.66 |
| Manaquiri | 212.98 |
| Manaus | 246.17 |
| Manicoré | 133.23 |
| Maraã | 255.34 |
| Maués | 173.74 |
| Nhamundá | 308.03 |
| Nova Olinda do Norte | 36.01 |
| Novo Airão | 289.96 |
| Novo Aripuanã | 264.94 |
| Parintins | 42.02 |
| Pauini | 113.99 |
| Presidente Figueiredo | 150.39 |
| Rio Preto da Eva | 198.57 |
| Santa Isabel do Rio Negro | 347.56 |
| Santo Antônio do Içá | 205.66 |
| São Gabriel da Cachoeira | 214.28 |
| São Paulo de Olivença | 168.81 |
| São Sebastião do Uatumã | 154.67 |
| Silves | 99.01 |
| Tabatinga | 146.05 |
| Tapauá | 241.85 |
| Tefé | 198.41 |
| Tonantins | 74.55 |
| Uarini | 235.03 |
| Urucará | 321.06 |
| Urucurituba | 9.55 |
