# Supplementary material for: Exploring the influence of deforestation on dengue fever incidence in the Brazilian Amazonas state
Source: PLoS One. 2021 Jan 7;16(1):e0242685. doi: 10.1371/journal.pone.0242685 (PMC7790412; doi:10.1371/journal.pone.0242685)
Supplement: S4 Table — (DOCX) [file pone.0242685.s004.docx]

| **S4 Table. Change in Dengue Incidence 1–5 Years After Deforestation Event** | | | | | |
| --- | --- | --- | --- | --- | --- |
| Municipality | Dengue incidence per 100,000 after deforestation event | | | | |
|  | 1 year | 2 years | 3 years | 4 years | 5 years |
| Alvarães | 0.00 | 16.12 | 163.59 | 66.46 | 50.68 |
| Amaturá | 0.00 | 0.00 | 0.00 | 0.00 | 0.00 |
| Anamã | 97.83 | 0.00 | 15.77 | 0.00 | 15.34 |
| Anori | 0.00 | 0.00 | 61.21 | 0.00 | 0.00 |
| Apuí | -58.37 | -42.88 | 59.74 | 0.00 | -27.18 |
| Atalaia do Norte | 11.80 | 11.51 | 0.00 | 0.00 | 10.75 |
| Autazes | -11.85 | 4.83 | 31.74 | -11.85 | -1.36 |
| Barcelos | -33.40 | -40.18 | -25.68 | -47.03 | 70.80 |
| Barreirinha | 6.63 | 0.00 | 6.52 | 6.47 | 0.00 |
| Benjamin Constant | 13.35 | 0.00 | 25.22 | 18.41 | 25.22 |
| Beruri | 12.75 | 0.00 | 0.00 | 0.00 | 11.99 |
| Boa Vista do Ramos | 13.19 | 0.00 | 0.00 | 0.00 | 0.00 |
| Boca do Acre | -86.79 | 367.29 | 144.94 | 203.31 | 338.86 |
| Borba | -5.96 | 12.83 | 12.16 | -35.50 | 117.86 |
| Caaparinga | 17.39 | 0.00 | 0.00 | 17.66 | 0.00 |
| Canutama | -32.70 | -16.89 | -32.70 | -32.70 | -17.37 |
| Carauari | -16.26 | -16.26 | -16.26 | -16.26 | -16.26 |
| Careiro | 193.05 | 68.25 | 119.02 | -0.19 | 16.69 |
| Careiro da Várzea | 11.71 | 2.67 | -13.92 | -10.11 | -6.50 |
| Coari | 81.85 | -52.06 | 431.68 | 204.31 | -86.75 |
| Codajás | 0.00 | 95.75 | 148.73 | 0.00 | 26.48 |
| Eirunepé | 0.00 | 0.00 | 0.00 | 0.00 | 0.00 |
| Envira | 0.00 | 0.00 | 0.00 | 0.00 | 0.00 |
| Fonte Boa | 29.94 | 0.00 | 0.00 | 0.00 | 0.00 |
| Guajará | 0.00 | 0.00 | 0.00 | 1460.76 | 1534.37 |
| Humaitá | 66.04 | 274.82 | 453.70 | 14.48 | 84.79 |
| Ipixuna | 28.36 | 27.73 | 13.56 | 19.92 | 19.92 |
| Iranduba | *NA* | *NA* | *NA* | *NA* | *NA* |
| Itacoatiara | -3.66 | 10.26 | 203.70 | 33.94 | 118.55 |
| Itamarati | 29.41 | 0.00 | 0.00 | 0.00 | 0.00 |
| Itapiranga | 61.80 | 63.25 | 0.00 | 0.00 | 0.00 |
| Japurá | 0.00 | 60.04 | 0.00 | 36.38 | 0.00 |
| Juruá | 0.00 | 0.00 | 0.00 | -20.59 | -20.59 |
| Jutaí | 73.64 | 0.00 | 0.00 | 0.00 | 0.00 |
| Lábrea | -1.65 | -2.52 | 24.10 | 23.91 | -129.15 |
| Manacapuru | -82.63 | 284.78 | 31.74 | 86.33 | -81.55 |
| Manaquiri | 4.94 | 0.00 | -23.83 | -1.57 | -23.83 |
| Manaus | 2575.01 | 101.84 | 642.46 | 581.85 | 509.29 |
| Manicoré | 3.43 | -0.12 | -175.76 | -255.09 | -135.61 |
| Maraã | 109.73 | 38.47 | 27.06 | 28.63 | 0.00 |
| Maués | 18.23 | 57.31 | 25.69 | -0.57 | 27.60 |
| Nhamundá | 0.00 | 0.00 | 0.00 | 0.00 | 0.00 |
| Nova Olinda do Norte | 51.52 | 11.22 | 16.50 | 5.40 | 79.45 |
| Novo Airão | 71.45 | 0.00 | 32.05 | 15.21 | 28.92 |
| Novo Aripuanã | 97.98 | 68.12 | 158.05 | 97.98 | 122.74 |
| Parintins | 0.01 | 0.01 | 0.02 | 3.15 | 22.00 |
| Pauini | 0.00 | 0.00 | 0.00 | 0.00 | 0.00 |
| Presidente Figueiredo | -57.48 | 47.93 | 283.49 | -0.37 | 48.03 |
| Rio Preto da Eva | -0.36 | -0.50 | 337.07 | -15.30 | -0.78 |
| Santa Isabel do Rio Negro | 0.00 | 0.00 | 0.00 | 0.00 | 0.00 |
| Santo Antônio do Içá | 0.00 | 151.16 | 11.71 | 12.75 | 0.00 |
| São Gabriel da Cachoeira | 65.60 | 114.92 | 65.79 | 82.45 | 27.58 |
| São Paulo de Olivença | 0.00 | 0.00 | 0.00 | 0.00 | 0.00 |
| São Sebastião do Uatumã | 0.00 | 20.92 | 0.00 | 0.00 | 0.00 |
| Silves | 0.00 | 0.00 | 22.69 | 0.00 | 0.00 |
| Tabatinga | -66.17 | 212.16 | -94.62 | 212.54 | 756.81 |
| Tapauá | 12.19 | 0.00 | 12.99 | 0.00 | 55.47 |
| Tefé | 37.33 | -85.71 | 96.17 | 555.58 | 319.22 |
| Tonantins | *NA* | *NA* | *NA* | *NA* | *NA* |
| Uarini | -22.30 | -22.30 | -22.30 | 0.00 | 22.30 |
| Urucará | 0.00 | 0.00 | 0.00 | 0.00 | 0.00 |
| Urucurituba | 13.26 | 0.00 | 0.00 | 0.00 | 12.23 |
